# Carbon Dots’ Antiviral Functions Against Noroviruses

**DOI:** 10.1038/s41598-017-00675-x

**Published:** 2017-03-31

**Authors:** Xiuli Dong, Marsha M. Moyer, Fan Yang, Ya-Ping Sun, Liju Yang

**Affiliations:** 1 Biomanufacturing Research Institute and Technology Enterprise (BRITE) and Department of Pharmaceutical Sciences, North Carolina Central University, Durham, NC 27707 USA; 2 Department of Chemistry and Laboratory for Emerging Materials and Technology, Clemson University, Clemson, SC 29634 USA

## Abstract

This study reported the first assessment of carbon dots’ (CDots) antiviral activity to human norovirus virus-like-particles (VLPs), GI.1 and GII.4 VLPs. CDots with different surface passivation molecules, 2,2′-(ethylenedioxy)bis(ethylamine) (EDA)-CDots and 3-ethoxypropylamine (EPA)-CDots, were synthesized and evaluated. The results indicated both EDA- and EPA- CDots were highly effective to inhibit both strains of VLPs’ bindings to histo-blood group antigens (HBGA) receptors on human cells at CDots concentration of 5 µg/mL, with EDA-CDots achieving 100% inhibition and EPA CDots achieving 85–99% inhibition. At low CDots concentration (2 µg/mL), positively charged EDA-CDots exhibited higher inhibitory effect (~82%) than non-charged EPA-CDots (~60%), suggesting the surface charge status of CDots played a role in the interactions between CDots and the negatively charged VLPs. Both types of CDots also exhibited inhibitory effect on VLP’s binding to their respective antibodies, but much less effective than those to HBGA binding. After CDots treatments, VLPs remained intact, and no degradation was observed on VLPs’ capsid proteins. Taken together, the observed antiviral effects of CDots on noroviruses were mainly through the effective inhibition of VLPs’ binding to HBGA receptors and moderate inhibition of VLPs’ binding to their antibodies, without affecting the integrity of viral capsid protein and the viral particle.

## Introduction

Human Norovirus (NoV) is the most common cause of nonbacterial, acute gastroenteritis outbreaks worldwide^1, 2^, accounting for more than 21 million illnesses and hospitalizations, and at least 570 deaths in the United States each year (Centers for Disease control and Prevention, 2013). NoVs are a group of related non-enveloped, single stranded RNA viruses that have been classified in the Calicivirdae family. NoVs contain six genogroups (from GI to GIV), which can be further divided into different genetic clusters or genotypes based on their capsid sequence^1^. For example, GI includes nine genotypes and GII contains 22 genotypes^1, 3^. Genogroups GI, GII, and GIV are responsible for disease in humans^4^.

NoV is extremely contagious and affects people of all ages. Human NoV transmission occurs by the fecal-oral route, usually through ingestion of contaminated food or water^5^, by breathing the air near an episode of vomiting, or by direct contact with an infected individual (62–84% of all reported outbreaks). NoV aerosols are formed during vomiting. A single episode of vomiting could release as many as 30 million virus particles^6^, while fewer than twenty virus particles can cause an infection^7^. NoV aerosols can also be formed by toilet flushing when vomit or diarrhea is present. The large amount of virus releasing from both fecal material and vomitus of infected individuals and the low infectious dose threshold are the factors that lead to the high number of human NoV annual outbreaks.

Studies have shown that NoVs recognize and interact with human histo-blood group antigens (HBGAs) in intestinal tissues as receptors or attachment factors in a strain-specific manner^8, 9^. HBGAs are complex carbohydrates and represent terminal structures of glycan chains. They are highly polymorphic and include three major families: the ABO, secretor, and Lewis families. HBGAs are presented abundantly on the surface of mucosal epithelia of gastrointestinal track, where they may function as anchors for NoVs to initiate an infection^10^. Previous studies suggested that synthetic HBGAs or HBGA-expressing enteric bacteria could enhance NoV infection in B cells^11^.

The prevention and control of human NoVs infections have been challenging, despite the more significant effort in recent years based on different chemical and physical antiviral methods^12–20^. Most of these methods have been extensions of their antibacterial uses, whereas NoVs are known to be resistant to commonly used sanitizers and disinfectants^21^. Among the more recently developed alternative antiviral strategies, the use of nanoparticles has yielded promising results, including for example silver nanoparticles^22^, gold-copper core-shell nanoparticles^23^, and TiO_2_ nanoparticles coupled with illumination of low-pressure UV light^24^.

A major difficulty in the study of human NoVs in general has been due to challenges in the cultivation of the virus *in vitro*, despite some progress very recently^25^, and to a lack of good animal model. Much of the research effort on NoVs has been based on the use of cultivatable surrogates such as murine norovirus, feline calicivirus, and poliovirus^16, 26^ and more conveniently the virus-like-particles (VLPs). VLPs are self-assembled VP1 capsid proteins, which are expressed from open read frame 2 (ORF2) as a recombinant protein independent of other viral components. Each VLP is ~38 nm in diameter. While the VLPs do not contain the genomic RNA and are replication deficient, their structural and antigenic characteristics are indistinguishable from the native virion^27, 28^. The NoV VLPs have been used as a promising vaccine platform for their ability to elicit a strong humoral and cellular immune response^29^. The characteristics of NoV VLPs and the easy production systems make them appropriate models for studying NoVs in biological assays and for understanding some specific questions about human NoVs. For example, VLPs were used successfully as a model in our previously reported study on the antiviral activity of gold-copper core-shell nanoparticles^23^. They have also been used as a model system for studying many other chemical and physical antiviral methods^23^. They are also useful in modeling virus-cell interactions^27, 28^, and in identifying NoV binding receptors on human cells such as HBGAs^30^. In the work reported here, we used NoV VLPs as a model of human NoVs to explore the potential antiviral functions of the recently developed carbon dots.

Carbon dots (CDots)^31^ are small carbon nanoparticles with surface passivation, for which more effective has been the chemical functionalization of organic molecules^32, 33^. As a new class of quantum dot-like nanomaterials, CDots possess properties of bright fluorescence, no toxicity *in vitro* and *in vivo*, environmentally benign, simple synthetic routes, as well as photocatalytic functions resembling those found in conventional nanoscale semiconductors^34–36^. Many potential applications of CDots are being pursued across many fields such as chemical and biological sensing, bioimaging, nanomedicine, photocatalysis, and electrocatalysis^34^. Among the unique properties of CDots that are more relevant to the study reported here is the photo-activated antimicrobial function^36, 37^. In fact, CDots with visible light illumination were highly effective in the inhibition of *E. coli* cell activities in several experimental settings, which has been attributed mechanistically to the photodynamic effect in CDots. Interestingly and surprisingly, we found in this study the significant antiviral activity of CDots toward NoV VLPs. More specifically, effects of the CDots on VLPs’ HBGA binding, antibody binding, and on the integrity of capsid protein and intergrity of VLPs particles were examined. Mechanistic implications of the results are discussed.

## Materials and Methods

### Human NoV VLPs and antibodies

Human NoV GI.1 VLPs and GII.4 VLPs, and their respective monoclonal antibodies (anti-GI.1 VLP antibody mAb3901 and anti-GII.4 VLP antibody NS14), were generously provided by Dr. Robert Atmar’s laboratory at Baylor College of Medicine (Houston, TX). The secondary antibody used in ELISA tests was goat anti-mouse IgG H&L antibody conjugated to horseradish peroxidase (HRP), which was purchased from Abcam (Cambridge, MA). The secondary antibody used in western blot assays was goat anti-mouse antibody labeled with IRDye^®^ 800CW, which was purchased from LI-COR Biosciences (Lincoln, NE).

### CDots

**Figure 1:**
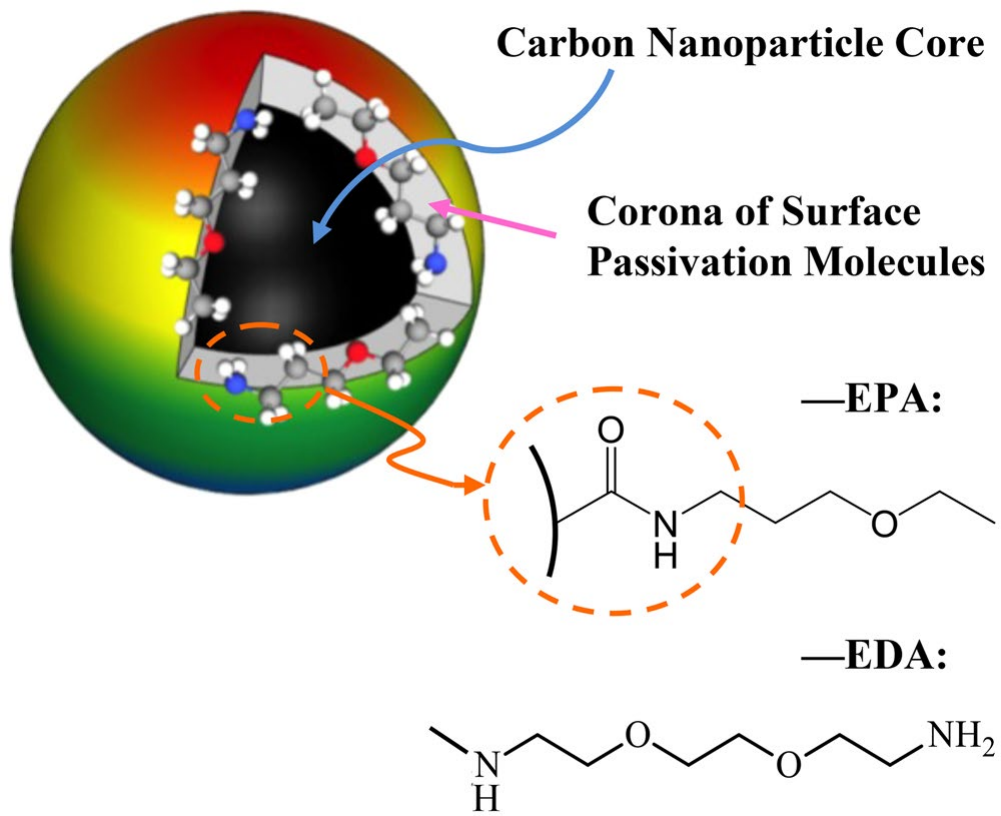
Schematic structure of a CDot. It consists of a carbon nanoparticle core and a thin layer of attached surface passivation molecules (EDA and EPA molecules specifically in this study).

The CDots were synthesized by chemical functionalization of small carbon nanoparticles, which were harvested from the commercially acquired carbon nano-powders (US Research Nanomaterials, Inc.) in procedures similar to those reported previously^37, 38^. In a typical experiment, a sample of carbon nano-powders (2 g) was refluxed in aqueous nitric acid (8 M, 200 mL) for 48 h. The reaction mixture was cooled back to room temperature, and centrifuged at 1,000* g* to discard the supernatant. The residue was re-dispersed in deionized water, dialyzed in a membrane tubing (molecular weight cut-off ~500) against fresh water for 48 h, and then centrifuged at 1,000 *g* to retain the supernatant. Upon the removal of water, small carbon nanoparticles were recovered and used in the functionalization reaction with 2,2′-(ethylenedioxy)bis(ethylamine) (EDA, Sigma-Aldrich)^37^ or 3-ethoxypropylamine (EPA, TCI America)^38, 39^ to yield EDA-CDots or EPA-CDots, respectively (Fig. 1).

For the synthesis of EDA-CDots^37, 39^, the small carbon nanoparticles were refluxed in neat thionyl chloride for 12 h. Upon the removal of excess thionyl chloride, the treated sample (50 mg) was mixed well with carefully dried EDA liquid in a round-bottom flask, heated to 120 °C, and vigorously stirred under nitrogen protection for 3 days. The reaction mixture back at room temperature was dispersed in water and then centrifuged at 20,000 *g* to retain the supernatant. It was dialyzed in a membrane tubing (cutoff molecular weight ~500) against fresh water to remove unreacted EDA and other small molecular species to obtain EDA-CDots as an aqueous solution. The EPA-CDots were synthesized in the same procedures under similar experimental conditions, as reported recently^38, 39^. Both EDA-CDots and EPA-CDots were characterized by using NMR, microscopy, and optical spectroscopy techniques, from which the results are consistent with the dot structure illustrated in Fig. 1. According to atomic force microscopy (AFM) and transmission electron microscopy (TEM) results, both EDA-CDots and EPA-CDots were size-wise 4–5 nm in average diameter, with the latter being on average slightly smaller^37, 38^.

### Saliva-based histo-blood group antigen (HBGA) receptor binding assay to evaluate the effect of CDots treatment on VLPs’ HBGA binding capacity

The effect of CDots treatment on VLPs was evaluated using a saliva-based HBGA binding assay according to a protocol reported previously with minor modifications^8, 9^. Briefly, saliva samples from healthy adult volunteers, including blood type A, B, and O, were collected. Saliva samples were immediately boiled for 5 min and centrifuged at 10,000 *g* for 5 min. The supernatant was collected and diluted to 1:2000 in PBS. For coating the plates with HBGAs, aliquot of 50 µL saliva dilution was used to coat 96-well plates at 4 °C overnight. Unbound saliva was removed and the wells were rinsed three times with PBS. The plates were then blocked with 100 µL Super-Block T20 (PBS) Blocking Buffer (Thermo Scientific Inc., Waltham, MA) for 1 h and rinsed with PBS twice.

For the HBGA binding assay, aliquots of 50 µL of 1.5 µg/mL VLPs, which were pretreated with 2 or 5 µg/mL EDA- or EPA- CDots at room temperature for 15 min, were added into the wells and incubated 1 h at room temperature. The unbound VLPs were removed and the wells were rinsed with PBST twice. The GI.1 or GII.4 VLPs binding to HBGAs in the wells were detected by using 1 µg/mL primary antibody mAb 3901 (to GI.1) or mAb NS14 (to GII.4), respectively, followed by the addition of 0.5 µg/mL secondary antibody–HRP-labeled goat anti-mouse IgG antibody. The antibody binding conditions included 1 h incubation at 37 °C and rinse with PBST twice. The final products were developed by adding 3,3′,5,5′-tetramethylbenzidine (TMB) peroxidase substrate (KPL, Gaithersburg, MD), and the absorbance reading at the wavelength of 450 nm was performed using a Spectra-Max M5 plate reader (Molecular Devices, Sunnyvale, CA).

### ELISA test to evaluate the effect of CDots treatments on human NoV VLPs’ antibody binding capacity

EDA- and EPA- CDots with various concentrations ranging from 0 to 60 µg/mL were used to treat 1 µg/mL GI.1 or GII.4 VLPs in medium-binding 96-well polystyrene plates (Costar^#^3591; Corning Incorporated, Corning, NY). 1 × PBS was added to reach the final volume of 50 µL in each reaction. The plates were constantly agitated on the shaker at the setting level of 2 at room temperature for 30 min, followed by 30 min incubation without agitation. The reaction solutions were discarded and the wells were washed with 100 µL 1 × PBS twice. The wells were then blocked with 100 µL Super-Block T20 (PBS) Blocking Buffer (Thermo Scientific Inc., Waltham, MA) for 1 h. After the blocking solution was discarded, each wells was washed with 100 µL PBST twice. Then, aliquots of 50 µL of 1 µg/mL anti-GI.1 VLP antibody mAb 3901 or anti-GII.4 VLP antibody mAb NS14 were added to each well to bind with the bound GI.1 or GII.4 VLPs, respectively, followed by 1 h incubation at 37 °C. After wash with PBST twice, aliquot of 50 µL of 1 µg/mL HRP-labeled goat anti-mouse IgG antibody solution was added to each well, and the plates were incubated at 37 °C for 1 h. After the incubation, the plates were washed with PBST, and the final products were developed using the TMB kits, and the absorbance at 450 nm in each well was measured.

### SDS-PAGE and western blotting for evaluation of the effect of CDots on VLPs capsid protein

EDA- and EPA- Cdots at the concentration of 20 or 60 µg/mL were used to treat 33.3 µg/mL GI.1 or GII.4 VLPs in 1.5 mL centrifuge tubes. 1 × PBS was added to reach the final volume of 12 µL in each reaction. The tubes were constantly agitated on a shaker (Lab-Line instruments Inc., Melrose Park, IL) at the setting level of 2 at the room temperature for 30 min. After the CDots treatments, each tube was added with 5 µL of 1 × NuPAGE LDS Sample buffer (Thermo Fisher Scientific, Wltham, MA), 2 µL of 1 M DTT, and 3 µL deionized water (DI-H_2_O). All the samples were incubated at 70–80 °C for 10 min and then loaded on 2 of precast 1.0 mm × 10-well NuPAGE® 4–12% Bis-Tris gels (Life Technologies, Grand Island, NY). The loading volume was 10 µL for each well. The gels were run in 1 × MOPS SDS running buffer (Invitrogen, Carlsbad, CA) at 200 V for 1 h. One gel was used for staining, while the other was for western blotting. The gel for staining was prefixed with a 50% methanol and 7% acetic acid solution for 15 min and then washed with DI-H_2_O for 5 min, three times. The GelCode Blue stain (Pierce Biotechnologies, Rockford, IL) was used to stain the gel with constantly shaking for 1 h, followed by 1 h de-staining step in DI-H_2_O. The gel was then imaged using a LI-COR Odyssey Infra-red Imaging System (LI-COR Biotechnology, Lincoln, NE).

For western blotting, the gel was transferred to an Odyssey® nitrocellulose membrane (LI-COR Biotechnology, Lincoln, NE) using 1 × NuPAGE® Transfer Buffer plus 10% MeOH and Hoefer Semi-Dry Transfer Apparatus (Hoefer Inc., San Francisco, CA) at 25 V for 1 h. The membrane was then blocked with 10 mL of 1:1 blocking buffer (Rockland Immuno-chemicals Inc., Limerick, PA) and PBS at room temperature for 1 h. The primary antibody treatment was performed by soaking the membrane in 10 mL of 1:1 PBST and blocking buffer, to which 4.11 µg of mouse monoclonal anti-GI.1 VLP antibody mAb 3901 or anti-GII.4 VLP antibody mAb NS14 had been added, followed by incubation at 4 °C overnight with gently shaking. Then, the antibody solution was discarded, and the membrane was washed 5 times, each with PBS plus 0.05% Tween 20 (PBST) for 5 min, and then treated with 0.5 µg of goat anti-mouse IRDye® 800CW antibodies in 10 mL of 1:1 PBST and blocking buffer at room temperature for 1 h. After washed 5 × 5 min with PBST under shaking, the membrane was soaked in DI-H_2_O and then imaged using the LI-COR Odyssey Infra-red Imaging System.

### Transmission electron microscopic (TEM) imaging

GI.1 and GII.4 VLP samples were treated with or without CDots, then 10 µL of each sample was placed on a formvar/carbon TEM grid (Electron Microscopy Sciences, Hatfield, PA) for 30 min. All grids were gently wicked to remove the fluid on the surfaces by the use of filter paper. The grids were stained with 2% uranyl acetate for 60 s and TEM images were acquired using a FEI Technai G2 Twin TEM (Hillsboro, OR) in the Shared Materials Instrumentation Facility (SMIF) at Duke University.

## Results and Discussion

### Inhibitory effect of CDots on VLPs’ binding to HBGA receptors

NoVs recognize human HBGAs as receptors or attachment factors, such binding events play an important role in host susceptibility to NoV infection^40, 41^. The binding of norovirus to HBGAs has been found to be highly diverse but strain-specific. Several binding patterns have been identified and grouped into two major binding groups based on the binding of 14 novrovirus strains to HBGAs, and a model of norovirus/HBGA binding has been proposed^9^. A retrospective study showed that type O individuals had a significant higher infection rate than those with other blood types^30^, while other studies showed Norwalk VLPs lacked the binding to saliva samples collected from nonsecretors, and saliva from type B individuals did not bind or weakly bound to Norwalk virus^42^. Therefore, in this study, we examined the effect of CDots on GI.1 and GII.4 VLPs’ binding to saliva HBGAs from blood Type A, B, and O.

**Figure 2:**
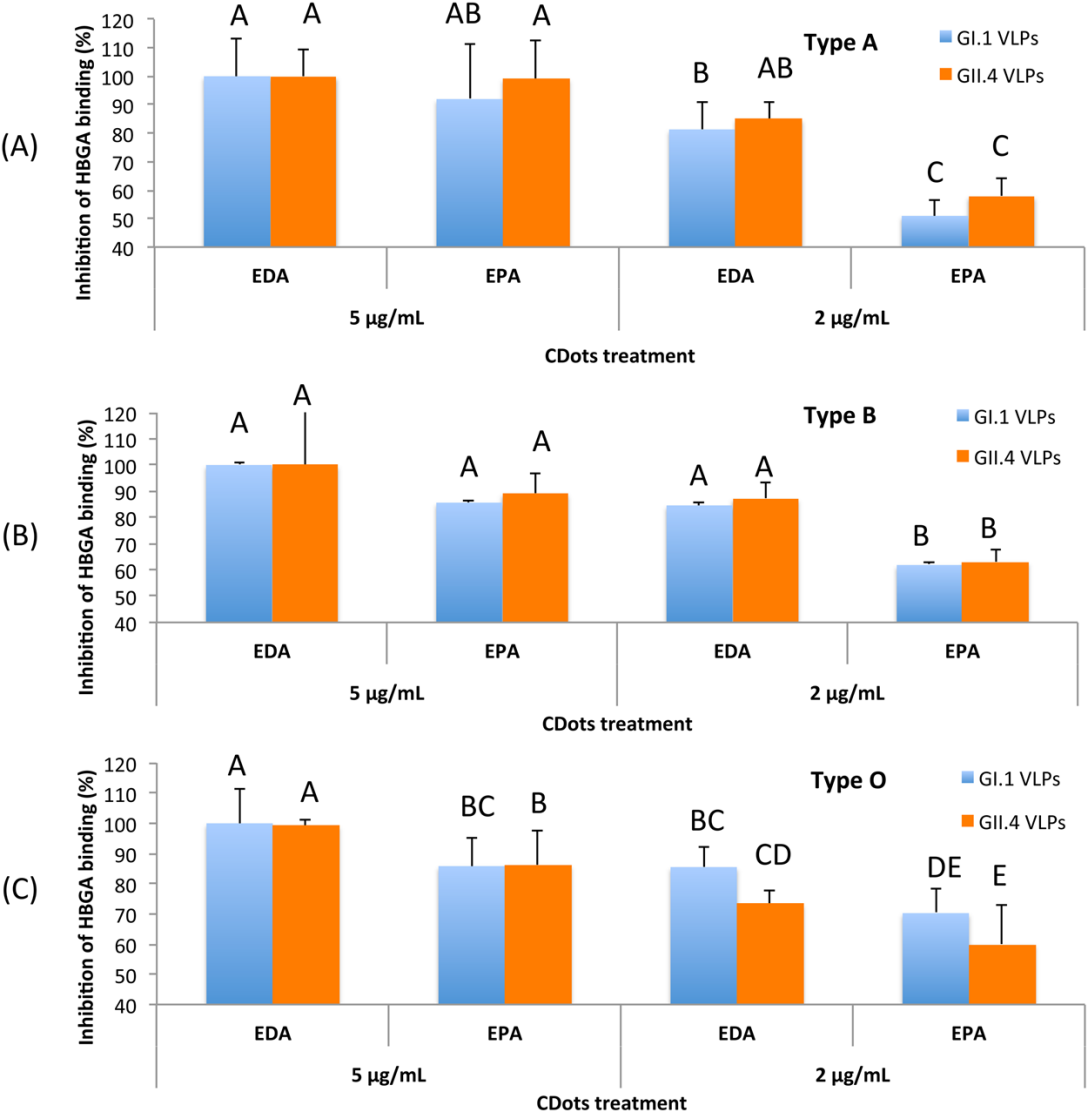
The inhibitory effects of CDots on NoV VLPs’ binding to type A, B, O, saliva HBGA receptors. (**A**) Type A HBGA; (**B**) Type B HBGA; (**C**) Type O HBGA. Different letters on the columns indicate significant differences at P < 0.05, while any same letters on the columns indicate no significant difference.

Figure 2 shows the inhibition percentages of VLPs’ binding to type A, B, O HBGA receptors by the treatments with EDA- and EPA-CDots, calculated using the untreated samples as the controls (100% binding). Figure 2A shows the inhibition effects of the treatment with the two types of CDots to the two strains of VLPs on their binding to type A HBGA receptors. For GI.1 VLPs treated with EDA-CDots at 5 µg/mL, the bindings to type A saliva HBGA receptors were completely inhibited (100% inhibition, Fig. 2A), indicating highly efficient inhibition effect of EDA-CDots on GI.1 VLP’s binding to HBGA receptors. The same quantitative inhibition (100%) was observed in GII.4 VLP bindings to the type A HBGA receptors with the treatment of 5 µg/mL EDA-CDots (Fig. 2A). The inhibitory effect remained strong even at lower CDot concentrations, such as the more than 80% inhibition in bindings of both GI.1 and GII.4 VLPs with the treatment of 2 µg/mL EDA-CDots (Fig. 2A). The results also suggested that the inhibitory effect of EDA-CDots on HBGA receptor binding was consistent between the different strains of VLPs.

EPA-CDots were also highly effective in the same inhibition, though relatively somewhat weaker on the marching concentration basis. As also shown in Fig. 2A, the treatment with EPA-CDots of 5 µg/mL and 2 µg/mL resulted in 91% and ~51% inhibition on GI.1 VLPs’ binding to type A HBGA receptors, respectively. Similar inhibition effect of EPA-CDots treatment to GII.4 VLPs was observed (Fig. 2A). According to the results shown in Fig. 2B and C, the inhibition effects of EDA- and EPA-CDots to the two strains of VLPs on their bindings to type B and type O HBGA receptors were equally strong. The dot concentration dependence and the difference between the two types of CDots were similar to those found in the inhibition to type A HBGA receptors shown in Fig. 2A.

The results presented above suggested that EDA-CDots were more effective than EPA-CDots in inhibiting VLPs’ binding to all three types of HBGA receptors for the two different strains of VLPs. The different effectiveness may be attributed to the different surface charge status and hydrophobicity property between the two types of CDots. EDA-CDots with the surface amino (-NH_2_) terminal groups tend to be positively changed at physiological pH (-NH_3_^+^), whereas EPA-CDots with surface methyl (-CH_3_) terminal groups are not charged. As VLPs are negatively charged, they should be more attractive to the positively charged EDA-CDots, resulting in a higher “local concentration” of the dots around the VLP particles, even though the mechanistic details on the interactions of the CDots with VLPs and the associated inhibition effects are likely very complex^9, 11^. On possible mechanisms for the observed strong inhibitory effects of CDots, one is such that the CDots would bind to the surface of VLPs and physically block the active sites on the VLPs used for bindings to the HBGA receptors. Based on the X-ray crystal structure on the prototype GI.1 of NoV^43^, it contains two domains: the shell (S) and the protruding (P) domain, and the HBGA receptor binding interfaces are located at the top of the P domain, containing carbohydrate binding pockets. These pockets involve several scattered amino acid residues that form extensive hydrogen bond network with individual saccharides, thus stabilizing the binding of HBGAs to the virus capsid protein^44, 45^. Although the binding of norovirus with human HBGA is a typical protein-carbohydrate interaction in which the protruding domain of the viral capsid protein serves an interface for the oligosaccharide side-chains of the HBGAs^40^, some of the complexities in the HBGA binding interactions have been discussed in the literature^11^, including capsid P domain loop movements, alternative HBGA conformations, and HBGA rotations. In fact, the blocking of NoV HBGA binding sites has been used as a surrogate for a NoV neutralization assay by using sera from immunized animals or infected humans^46, 47^. It was found that the ability of sera to block VLP-HBGA interactions could be correlated with the protection against infection in NoV-vaccinated chimpanzees and against the illness among infected human volunteers^48, 49^. According to these reported studies, the blocking of the HuNoV capsid from recognizing their binding sites on host cells represents a promising strategy in preventing HuNoV infection. Thus, the observed effective inhibition of the NoV VLPs by the CDots (Fig. 2) may be considered as an application of such a strategy.

Interactions between various carbon nanomaterials and proteins in different mechanisms have been well-documented in the literature^50, 51^, as relevant to the expected interactions of CDots with VLPs capsid proteins. For example, it is known that carbon nanotubes (CNTs) can nonspecifically bind to proteins through complementary charges, π-π stacking, and/or hydrophobic interactions^52^. Analyses of the binding between C_60_ (a special type of carbon nanoparticles) and lysozyme revealed that the primary driving force for the binding is van der Waals interaction, while polar solvation and entropy are detrimental to the binding^53^. More relevant to the blocking of receptor sites, it was demonstrated that C_60_ could inhibit the activity of HIV-proteases by integrating with proteins to form hybrid functional assemblies^54^. Therefore, a conceptually analogous explanation on the observed inhibition of NoV VLPs might be such that the CDots interact with VLPs’ capsid protein by a combination of several driving forces, resulting in the blocking of the active sites on NoV VLPs responsible for their binding to HBGA receptors.

### Inhibitory effects of CDots on VLPs’ binding to their antibodies

**Figure 3:**
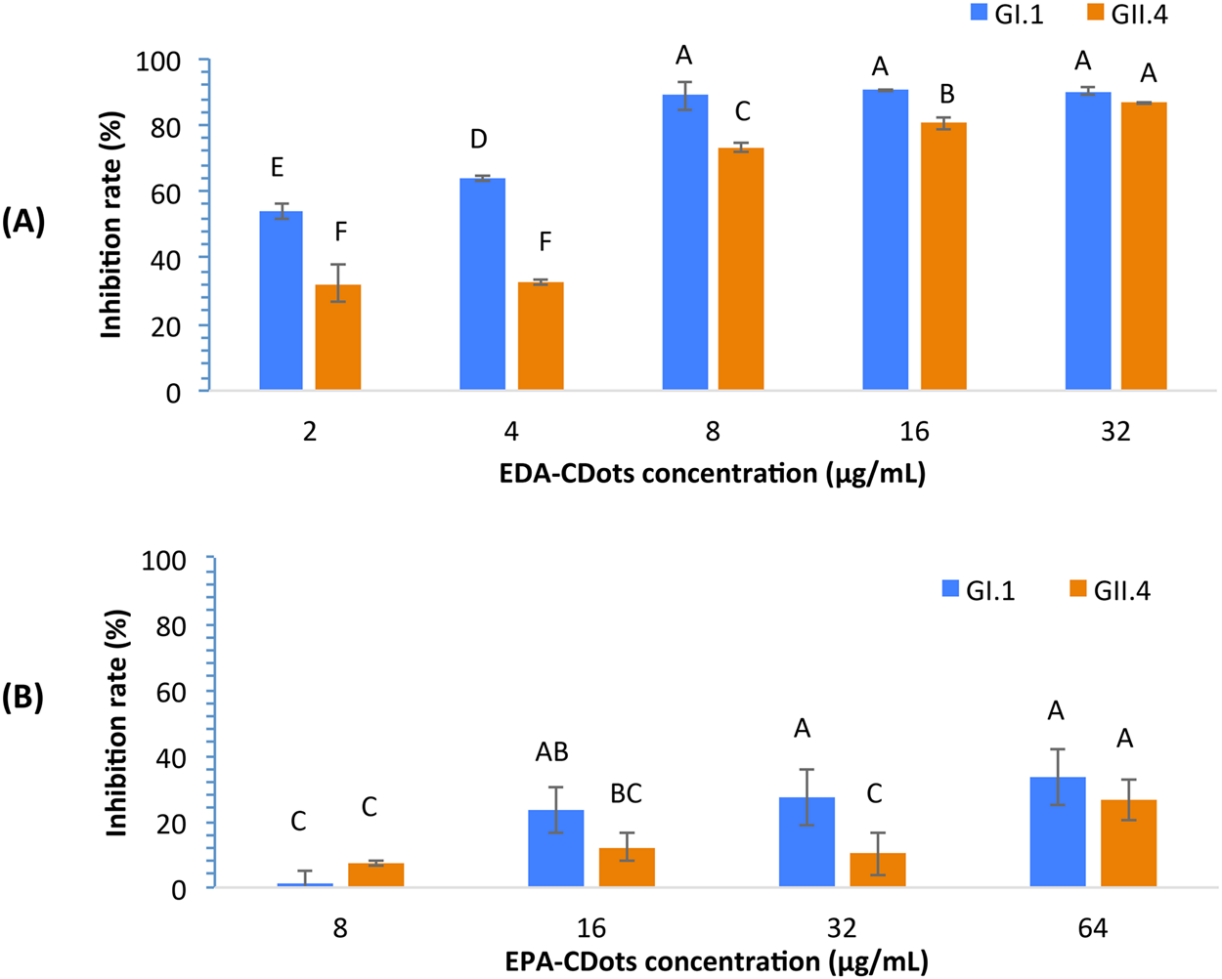
Inhibition effects of EDA-CDots and EPA-CDots on GI.1 and GII.4 VLPs’ binding to their respective antibodies. (**A**) EDA-CDots and (**B**) EPA-CDots. Statistic analysis was performed using SAS 9.2. Different letters on the columns indicate significant differences at P < 0.05, while the same letters indicate no significant difference.

We further examined the effect of CDots treatment on VLPs’ binding to their antibodies. Figure 3A shows the inhibition percentages of EDA-CDots treatment to both GI.1 and GII.4 VLP’s binding activities to their antibodies (mAb 3901 for GI.1 and mAb NS14 to GII.4) at dot concentrations of 0 to 32 µg/mL. For 2 µg/mL EDA-CDots as an example, GI.1 and GII.4 VLP’s binding activities to their respective antibodies were inhibited by ~54% and ~32%, respectively. At a higher EDA-CDots concentration of 8 µg/mL, GI.1 and GII.4 VLP binding activities to their antibodies were inhibited by ~88 and 73%, respectively. The inhibition effect was apparently saturated with respect to higher dot concentrations, as even at a much higher EDA-CDots concentration of 32 µg/mL, the inhibition percentages improved to 90% and 87% for GI.1 and GII.4 VLPs, respectively, but still not completely (100%). The results indicated that between the two strains of VLPs, EDA-CDots are somewhat more effective in the inhibition of GI.1 VLPs’ binding to its mAb 3901 antibody than the inhibition of GII.4 VLPs’ binding to its mAb NS14 antibody (Fig. 3A).

EPA-CDots were somewhat less effective in the inhibitory effect on both GI.1 and GII.4 VLPs’ binding to their respective antibodies, as compared in Fig. 3B. The treatment with 8 µg/mL of EPA-CDots only resulted in 1% and 7.3% of inhibition to GI.1 and GII.4 VLPs’ binding to their antibodies, much less than those achieved with EDA-CDots (Fig. 3A). Even with the high EPA-CDots concentration of 32 µg/mL, the inhibition percentages for the binding of GI.1 and GII.4 VLPs to their antibodies were only ~27% and ~10%, respectively. However, a further increase in the EPA-CDots concentration to 64 µg/mL, the inhibition percentages for GI.1 and GII.4 VLPs’ binding to their antibodies improved to ~33% and ~26%, respectively, showing no saturation effect. The obviously less effective inhibition by EPA-CDots than that by EDA-CDots may again be attributed to the different surface charge status between the two types of CDots, as similarly discussed above on the inhibition of the VLPs’ binding to HBGA receptors.

In both EDA- and EPA-CDots treatments, GI.1 VLPs were more effectively inhibited in their binding to mAb3901 antibodies than GII.4 VLPs’ bindings to mAb NS14 across the different CDots concentrations (Fig. 3A and B). This might be due to the capsid structure difference in the two strains of VLPs involving VLP-antibody interactions. Interestingly, however, no significant difference was observed in the CDots’ inhibitory effect on GI.1 and GII.4 VLPs’ bindings to HBGA receptors (Fig. 2). Nevertheless, in the literature the difference in capsid structure in NoV GI.1 and GII.4 was found to be a factor accounting for variations in some other antiviral methods. For example, GI.1 VLPs were found to be more vulnerable to high-pressure processing (HPP) than that of the GII.4 strain^55^, where the disruption of viral envelope and/or capsid structure, not the degradation of the viral protein or genome, was the primary mechanism of HPP^55^.

The treatment with CDots was clearly more effective in inhibiting VLPs’ binding to HBGA than to their antibodies. In a comparison of the results in Figs 2 and 3, the VLPs’ binding to HBGA was quantitatively 100% inhibited by 5 µg/mL EDA-CDots for both GI.1 and GII.4 VLPs versus even at a higher EDA-CDots concentration of 8 µg/mL for only ~70–90% inhibition of VLP’s binding to their respective antibodies. The difference in inhibition effectiveness was more substantial in the use of EPA-CDots, as 5 µg/mL of the dots could inhibit ~85–90% HBGA binding versus 64 µg/mL of the dots only inhibiting ~30% of VLPs’ binding to their antibodies. Again, the results in this study and others (X. F. Zhang *et al*., 2013) suggested that the CDots treatment may be a potential strategy to prevent the initial access or spread of NoV to humans by effectively inhibiting NoV binding to HBGA receptors.

**Figure 4:**
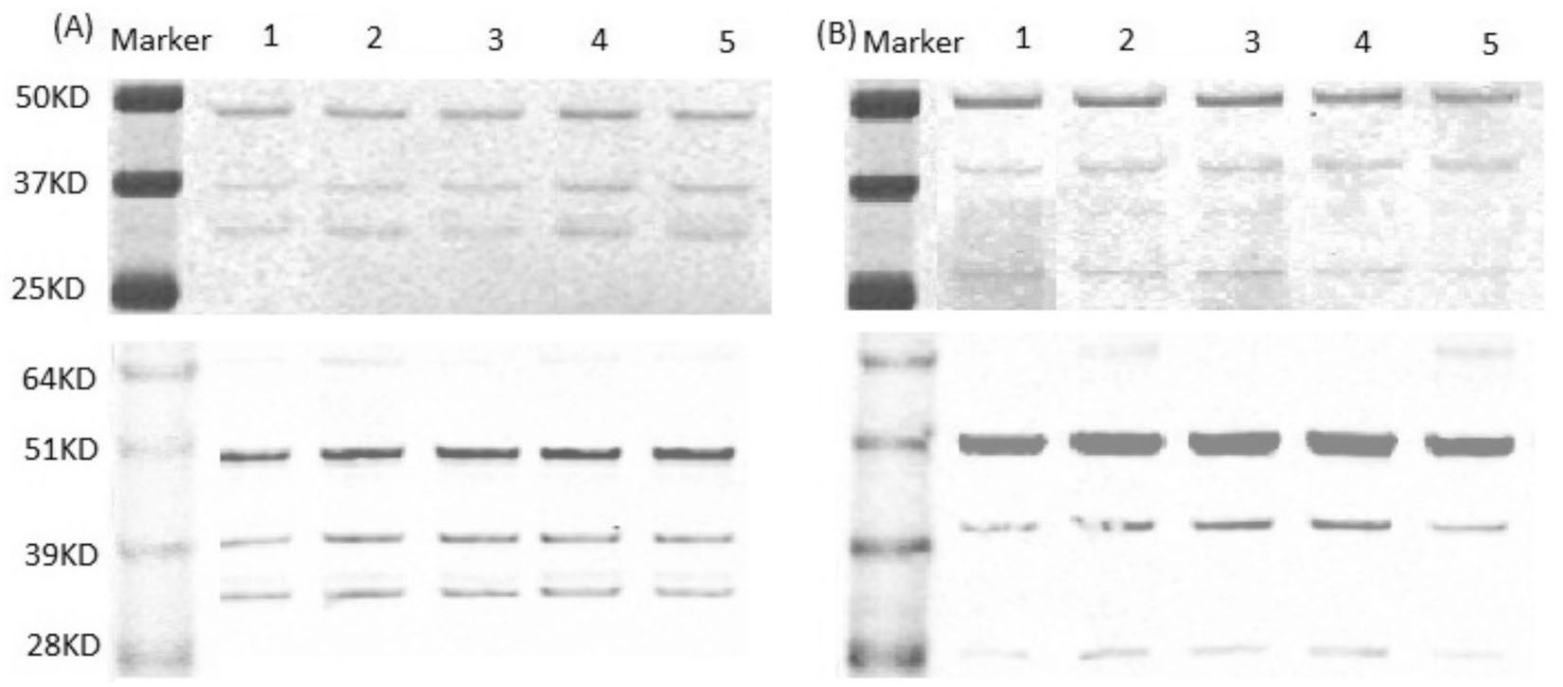
The results of SDS-PAGE gel (top) and Western blot (bottom) analysis of GI.1 VLPs (**A**) and GII.4 VLPs (**B**) after CDots treatments. Lane 1: VLPs control; Lane 2, 3: VLPs treated with 20 µg/mL of EDA-CDots and EPA-CDots, respectively; Lane 4, 5: VLPs treated with 60 µg/mL of EDA-CDots, and EPA-CDots, respectively.

### No degradation of VLPs’ capsid protein by CDots treatments

To gain some mechanistic insights into the inhibitory effect of CDots on VLPs’ binding capacity to their antibodies and HBGA receptors, we further examined the possibility of VLPs’ capsid proteins being degraded by CDots treatments. Briefly, GI.1 and GII.4 VLPs were treated with EDA- and EPA- CDots at two different doses (20 µg/mL and 60 µg/mL). The treated and untreated VLPs were analyzed by SDS-PAGE gel, followed by GelCode Blue staining. As shown in Fig. 4 (top), both GI.1 and GII.4 VLPs showed clear bands at ~50 KDa, likely the shifted bands of the full length capsid protein (VP1, ~58KDa), and the other bands at smaller sizes were most likely protein fragments from the capsid protein. Importantly, the protein band patterns and their abundance did not change after CDots treatments, even for the samples treated with 60 µg/mL CDots exhibiting no significant difference from untreated control samples, suggesting that VLPs’ capsid proteins were not degraded by CDots treatments, and VLP capsid protein structures remained intact.

We also examined the possibility of the proteins being antigenically changed after CDots treatments by western blotting using mAb 3901 against GI.1 VLPs and mAb NS14 against GII.4 VLPs. As shown in Fig. 4 (bottom), the abundance of VP1 and other protein fragments detected by western blotting in GI.1 and GII.4 VLPs did not change after CDots treatments. For the protein bands in GI.1 VLPs as an example, it is known that mAb 3901 can bind to either the full-length (58 KDa) capsid protein or a 32 KDa protein fragment in the P domain^56, 57^, recognizing a continuous epitope on the C-terminal of the capsid protein^57^. The antibody mAb 3901 also recognizes a domain between amino acid 453 and amino acid 495, and the lower band in the western blot is likely a fragment that contains this sequence. Similarly for GII.4 VLPs, the mAb NS 14 binds to the capsid protein and other protein fragments that contain the recognized epitopes. Clearly, for both GI.1 and GII.4 VLPs, the protein band patterns detected in western blotting were essentially the same as those observed in SDS-PAGE detected by GelCode Blue staining. Therefore, the results demonstrated that CDots treatments did not degrade the viral protein, as the viral proteins still retained correct primary amino acid sequences and were able to react with their antibodies.

### No damage on the integrity of VLP particles by CDots treatments

**Figure 5:**
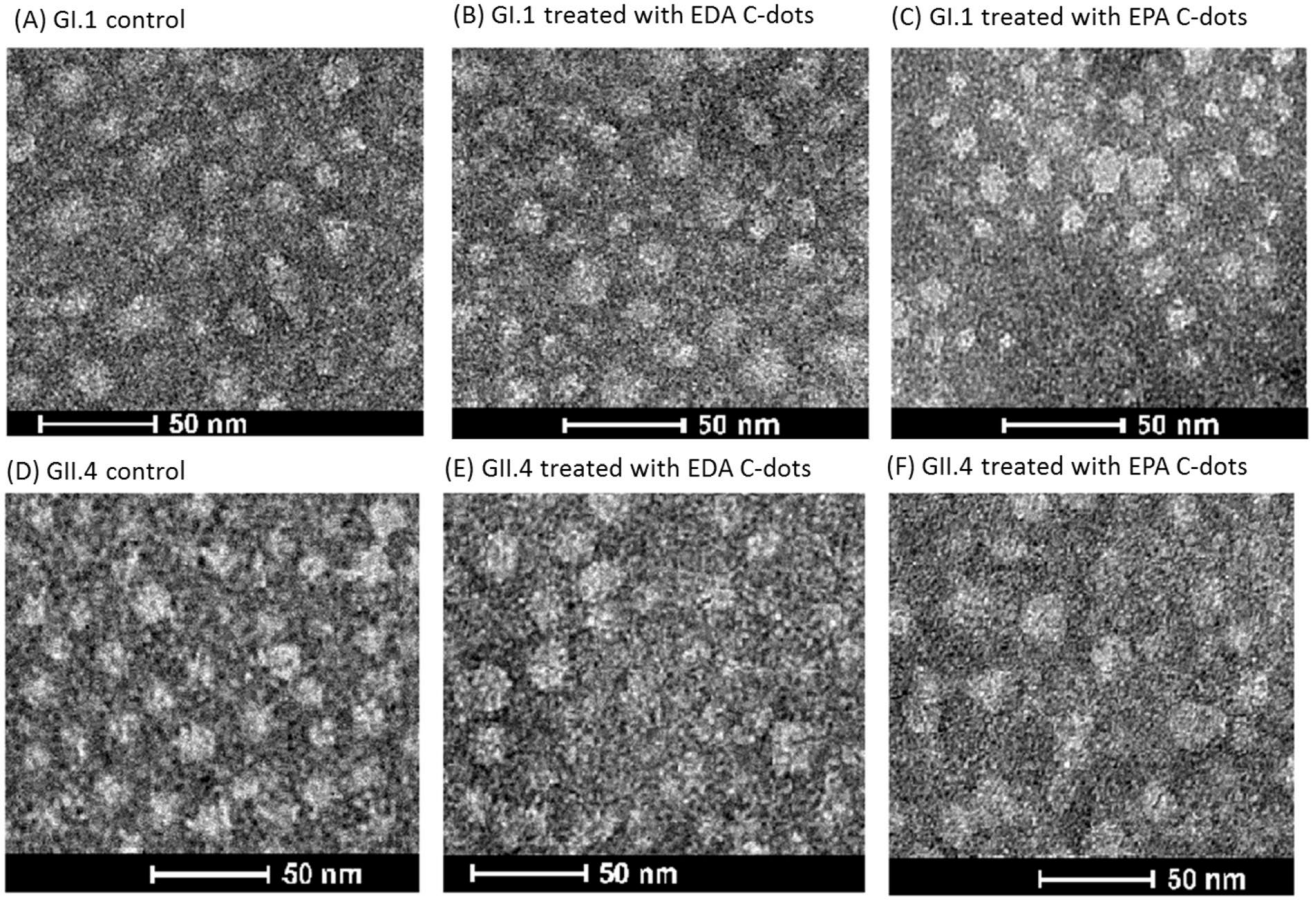
TEM images of GI.1 and GII.4 VLPs after different treatments. (**A,D**) Untreated, (**B,E**) Treated with EDA-CDots, and (**C,F**) Treated with EPA-CDots.

Shown in Fig. 5 are images of untreated GI.1 and GII.4 VLPs and the VLPs treated with EDA- and EPA-CDots. These images indicated that there were no changes in GI.1 and GII.4 VLP morphologies after CDots treatments, nor server aggregation of the VLPs. The observation might be expected for the fact that CDots have been studied extensively as fluorescence stain for cell imaging, causing no cell morphology changes. CDots are also known for their low to no cytotoxicity and high biocompatibility. For example, normal growths of zebrafish larvae were observed after their being soaked in 1.5 mg/mL CDots solution^58^. HeLa cell viability was over 90% after being incubated with 500 µg/mL of CDots for 24 h^59^. The observation that the morphology and integrity of VLPs remained unchanged after CDots treatments is consistent with these and other results reported in the literature.

## Conclusions

This study explored the effects of EDA-CDots and EPA-CDots on NoV GI.1 and GII.4 VLPs. The results demonstrated that the treatment with CDots effectively inhibited VLPs’ binding to saliva HBGA receptors (all three types A, B, and O), without degrading VLP capsid proteins or affecting the integrity and morphology of the VLP particles. Between the two types of CDots, the positively charged EDA-CDots were much more effective than the non-charged EPA-CDots in inhibiting the binding of VLPs to HBGA receptors, due to more favorable binding between EDA-CDots and negatively charged VLPs. These CDots also showed inhibitory effect on VLPs’ binding to their respective antibodies, but much less effective compared to those inhibitions to HBGA receptors. Nevertheless, the results from this study showed the proof-of-concept on CDots’ antiviral function through the inhibition of virus binding to HGBA receptors, which could be a promising strategy in preventing HuNoV infection/spread by disabling HuNoV recognizing their binding sites on host cells. As there is no effective vaccine for NoV, effective hand washing and cleaning of contaminated sites are recommended practices for prevention of NoV infection/spread, examples of potential applications include CDots-containing antiviral sprays for sanitizing NoVs contaminated sites, such as surfaces or instruments in hospital, patient vomiting on carpet/floor, bath room toilet after patient diarrhea. CDots can also be incorporated into routine hand wash soaps for antiviral purpose. For cases involving aerosol NoVs, incorporating CDots agents into air filtration devices may be explored. Though the practical application for such purpose is likely more complex and requires additional investigations, the reported initial assessment of CDots’ antiviral function is highly valuable, and will be significantly contributing to the knowledge matrix for the broad implications and application potential of CDots.
